# In-Situ Filtration Sampler for the Measurement of Trace Metals in Precipitation

**DOI:** 10.6028/jres.093.058

**Published:** 1988-06-01

**Authors:** Barbara J. Keller

**Affiliations:** Analytical Chemistry Unit, Illinois State Water Survey, Champaign, IL

An in-situ filtration sampler has been developed for use in measuring trace metals in precipitation. This sampler is a modification of the Aerochem Metrics Model 301 wet/dry precipitation sampler now in use in the National Atmospheric Deposition Program/National Trends Network (NADP/NTN). The sample is captured in a funnel and filtered directly into a precleaned and preweighed collection bottle. Upon collection, the bottle can be weighed to determine sample volume and immediately preserved with the appropriate amount of acid. Samples collected in this manner were compared to those collected in high density polyethylene (HDPE) buckets according the standard NADP/NTN protocols for major ion sampling. These bucket-collected samples were filtered in the laboratory at different times throughout a week to approximate time lapses normally present in a large scale monitoring study. The metals investigated were aluminum, cadmium, copper, iron, lead, manganese, and zinc. Graphite furnace atomic absorption spectrophotometry was the method of analysis.

Filtration of aqueous samples followed by acidification has been shown to be necessary to preserve the natural distribution of metals since the particles present in the sample may adsorb or desorb metal ions rapidly [1]. Immediate Filtration is particularly important in low volume deposition events where rapid pH changes can take place due to neutralization by natural dusts [2]. Since there are varying time lapses between collection in the field and filtration in the laboratory, the soluble/insoluble distribution of metals may be significantly changed in the final analysis. The in-situ filtration system partitions the sample as it falls, so that a more natural metal distribution is preserved. Also, sample handling is decreased, thereby decreasing opportunity for contamination.

The in-situ filtration system consists of a funnel and bottle assembly with an in-line filter. The top opening of the funnel is the same diameter as the standard buckets (30 cm), so that the catch area is the same. The HDPE funnel is connected to the fluorinated ethylene propylene (FEP) tubing via a polypropylene cone. The 0.4 *μ*m polycarbonate membrane filter is housed in a tetrafluoroethylene (TFE) filter holder. The samples are collected directly into a 2-liter polyethylene bottle. After collection, the bottle is weighed and the sample is preserved with the appropriate amount of ultrapure nitric acid.

The Aerochem Metrics Model 301 wet/dry precipitation sampler was modified as follows:

1. The counterweight bar was cut and the middle portion removed, so that tubing could be connected from the collection funnel to the receiving bottle.
2. The bucket was shortened by 2.5 cm (off the top) to allow the funnel to nest at the top of the bucket. This was necessary to allow full closure of the sampler lid during dry periods.
3. A hole was cut in the bottom of the bucket for passage of the polypropylene cone and FEP tubing.

Direct comparisons between this system and the bucket collection system involved blank leachates and natural wet deposition samples. Blank leachate results for the two systems are shown in table 1. Blanks were poured into the bucket or funnel, left for 24 hours, and transferred to sample bottles and preserved. Acid cleaning the buckets lowered the iron values, but the zinc values increased to as high as 115 *μ*g/L. The maximum concentrations found in the in-situ filtration system blank leachates were much lower for iron and zinc.

Figure 1 shows results for the 24-hour sampling period during which a rainfall of 0.35 inches was collected. The bucket sample was filtered and acidified at 0, 1, and 3 days, while the in-situ filtration sample was acidified at the same intervals. The differences are obvious with much higher aluminum, iron, and zinc concentrations in the bucket samples.

To check for adsorption of metals onto the collector and/or filter surfaces, spike recoveries were determined for the in-situ filtration system. Results are presented in table 2.

The gravity filtration system evaluated in this study has some limitations. Precipitation with a high particulate load may require a pumping system, and below freezing temperatures necessitate the use of a heating system and/or insulation. Further research into these areas is planned.

The advantages of in-situ filtration are: preservation of the natural distribution of metals, decreased sample handling, and the inexpensive modification of available equipment.

## Figures and Tables

**Figure 1:**
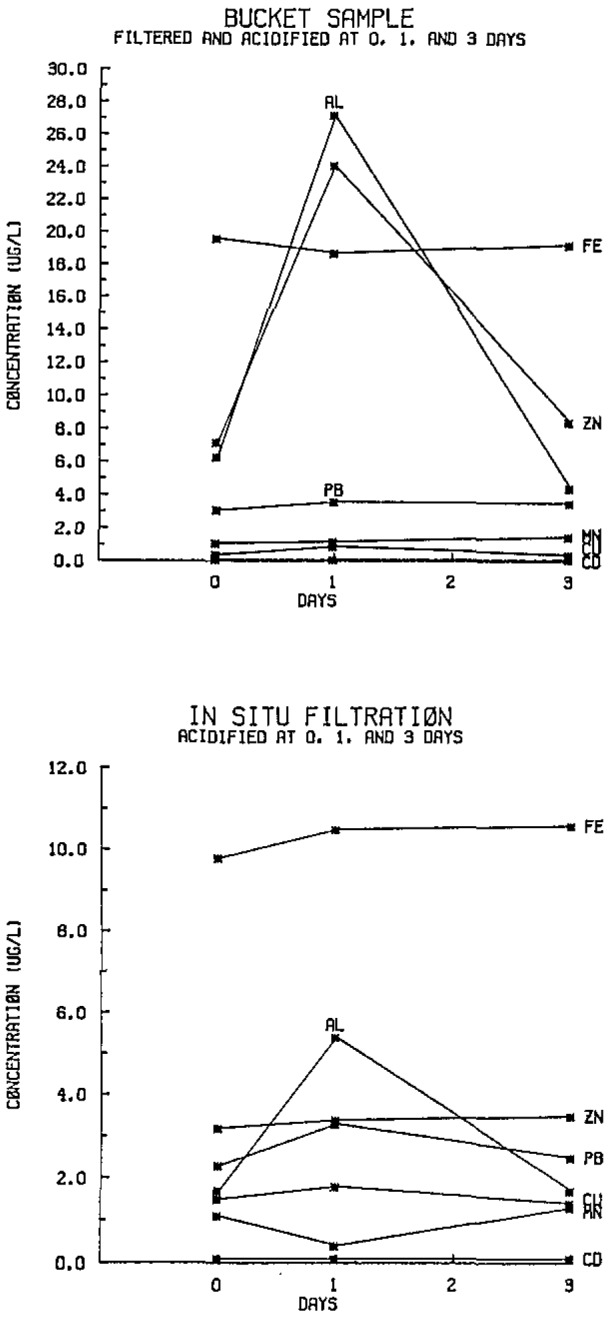
Trace metal concentrations of a rainfall of 0.35 inches, Champaign, IL, February 5, 1986.

**Table 1:** Blank leachate analyses for HDPE sampling buckets and the in-situ filtration sampling system (percent frequency of method detection limit (MDL) concentrations)

Metal	MDL (*μ*g/L)	HOPE buckets^a^	In-situ filtration system^b^
Al	3.5	100	82 (4.3)^c^
Cd	0.05	100	91 (0.08)
Cu	0.9	100	100
Fe	1.1	90 (16.1)	91 (1.2)
Pb	1.1	95 (2.8)	100
Mn	0.8	100	100
Zn	0.5	18 (14.7)	73 (2.9)

a pH 5.7 and pH 1.8, *n* =40

b pH 5.7, pH 4.3, and pH 3.4, *n* = 11

c Numbers in parentheses are maximum values (μg/L).

**Table 2:** Single-operator precision and bias for trace metals de termined from analyte spikes of samples (6 blanks, 2 synthetic, 5 wet deposition)^a^

Metal	Amount added, *μ*g/L	*n*	Mean percent recovery, %	Mean bias, *μ*g/L	Standard deviation, *μ*g/L	Statistically significant bias?^b^
Al	18.5	12	95.7	−0.8	2.1	no
Cd	6.11	13	109.2	0.56	0.73	yes
Cu	11.0	13	100.0	0.0	0.6	no
Fe	11.1	12	89.2	−1.2	0.9	yes
Pb	20.8	13	101.9	0.4	1.5	no
Mn	10.1	13	107.9	0.8	0.5	yes
Zn	21.9	13	107.8	1.7	4.7	no

a Samples were spiked prior to filtration in the in-situ filtration collector.

b 95% confidence level [3]
